# Barriers and Motivations to Provide Dental Care to Adult Patients with Movement Disorders

**DOI:** 10.3390/ijerph19095256

**Published:** 2022-04-26

**Authors:** Natalia S. Rozas, Hillary D. Strassner, June M. Sadowsky, Cameron B. Jeter

**Affiliations:** 1 Department of Diagnostic and Biomedical Sciences, School of Dentistry, The University of Texas Health Science Center at Houston (UTHealth), 7500 Cambridge Street, Suite 5456, Houston, TX 77054, USA; natalia.s.rozas@uth.tmc.edu; 2 Department of Pediatric Dentistry, School of Dentistry, The University of Texas Health Science Center at Houston (UTHealth), 7500 Cambridge Stree, Suite 5301, Houston, TX 77054, USA; hillary.strassner@uth.tmc.edu; 3 Department of General Practice and Dental Public Health, School of Dentistry, The University of Texas Health Science Center at Houston (UTHealth), 7500 Cambridge Street, Suite 5427, Houston, TX 77054, USA; june.sadowsky@uth.tmc.edu

**Keywords:** health services accessibility, movement disorders, dental care for aged, dentistry

## Abstract

Patients with movement disorders, like Parkinson’s and Huntington’s diseases, tend to have poor oral health. Although contributing factors have been proposed, the willingness and ability of dentists to treat this patient population are still unknown. Our objective is to understand the current barriers and motivations of dentists to treat this patient population as a path to improved care and quality of life. A total of 176 dentists in Texas were surveyed through a structured questionnaire which contained both closed and open-ended questions. Nearly 30% of participants reported having no barriers to treating these patients and 26.7% reported that no such patients have visited their practice. Barriers reported included lack of education on the topic (17.6%) and longer appointments than average (14.8%). A main motivation to treat these patients was more training and education on the subject (38.6%). Poor oral health in patients with movement disorders may not be due to barriers encountered by dentists, but rather encountered by patients, such as access to and use of dental treatment. General dentists are willing to provide care for adult patients with movement disorders and continuing education for these providers is preferred over referral to a specialist.

## 1. Introduction

Parkinson’s disease (PD) and Huntington’s disease (HD) are neurodegenerative disorders diagnosed primarily based on motor symptoms such as tremors, rigidity, and imbalance (PD), and uncontrolled movements (HD). PD is the second-most common neurodegenerative disorder after Alzheimer’s disease and has an incidence rate between 8 and 18 cases per 100,000 a year [1]. Patients suffer from neuronal loss in the basal ganglia region of the brain, caused by aggregated ⍺-synuclein protein in neuronal intracellular inclusions known as Lewy bodies [2,3,4]. In HD, an autosomal dominant disease, the gene that encodes the protein Huntingtin contains more repeats of the CAG nucleotide sequence. The abnormally long huntingtin protein aggregates and causes neuronal death in the basal ganglia [5]. The incidence rate of HD is between 5 and 13 cases per 100,000 a year [6]. Although best known for their motor symptoms, patients with PD or HD may also present non-motor symptoms such as autonomic dysfunction, depression, cognitive decline, and oral health problems [7].

Previous studies have shown that neurodegenerative disorders may affect the oral health status of patients and that poor oral health, in turn, may affect patients’ overall quality of life and disease progression in a bidirectional manner [8]. Patients with PD show a higher prevalence of caries, periodontal disease, and more abundant oral pathogens than healthy controls [9,10,11]. Very few reports have investigated oral health problems in patients with HD. One observational study and several case studies report more decayed teeth, frequent tooth loss, and a higher plaque index in these patients [12,13]. It is unknown why patients with movement disorders have poorer oral health than healthy controls, but several factors have been suggested: difficulty performing oral hygiene, medications that can cause dry mouth, missed dental appointments, motor symptoms that may complicate or prevent dental treatment, and cognitive problems that may impair communication and daily habits [7,14]. Because of the diversity of factors directly affecting patients’ ability to maintain their own oral health, education for dentists should not be limited to describing the oral symptoms of these patients, but also include how to support and coordinate individuals’ behavior to receive dental care.

Dental professionals have experienced and reported barriers when treating specific patient populations. When caring for older patients and/or geriatric patients living in long-term care, these barriers include lack of adequate dental equipment, lack of time or willingness to go to a patient’s home or residence, lack of work satisfaction and lack of adequate reimbursement [15]. Barriers reported for other patient populations, such as pregnant women, persons with disabilities, patients with mental illness, and patients with drug abuse, include lack of specialized training, difficulty communicating with the patient, lack of equipment, difficult appointments due to fear, prolonged appointment times, cost of treatment, and lack of equipment [16,17,18,19,20]. No study to date has investigated barriers encountered by dentists treating adult patients with movement disorders. Reducing these barriers and maximizing motivators could lead to better oral health care and better overall health for patients with movement disorders. Our objective was to investigate if dentists encounter barriers and/or motivators to aiding oral health in this patient population.

## 2. Materials and Methods

### 2.1. Sample and Data Collection

**Figure 1:**
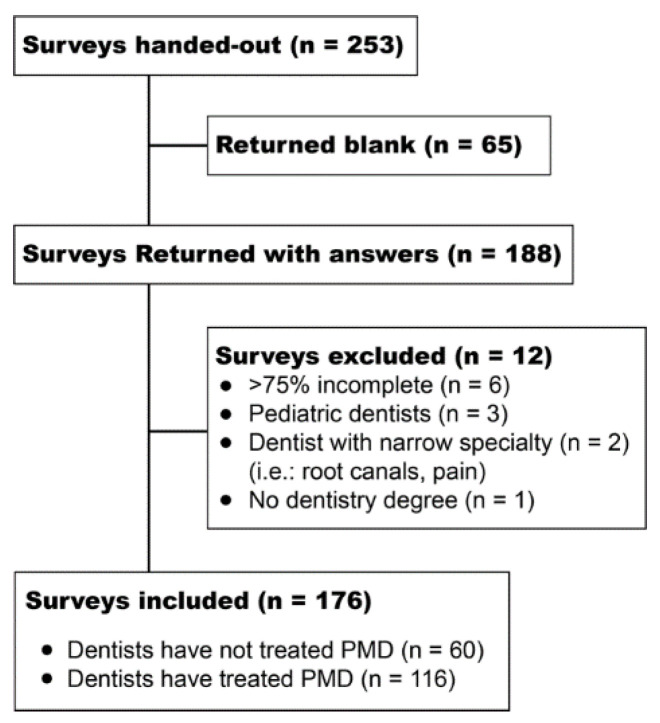
Flow chart of participant recruitment. PMD: patients with movement disorders.

The target population was general dentists with a minimum qualification of a Doctor of Dental Surgery (DDS) or Doctor of Dental Medicine (DMD) who were eligible to provide oral health care for patients with movement disorders. Dentists consecutively attending the Star of the South Dental Meeting in Houston, TX and continuing education courses at The University of Texas Health Science Center at Houston (UTHealth) School of Dentistry were approached and invited by a research assistant between 2015 and 2017 to participate in this study by completing a structured paper-based survey. Two hundred fifty-three dentists were invited and 188 consented and completed the survey; of those, 176 surveys met inclusion criteria and were included in the analysis (Figure 1). The inclusion criterion was dentists with a DDS or DMD licensed in the state of Texas. Exclusion criteria were dentists ineligible to treat adults with movement disorders (e.g., pediatric dentists) or who submitted a survey that was more than 75% incomplete. Dentists completed the survey in a room separate from the research assistant to protect privacy and reduce bias. No incentive or compensation was offered to the participants for completing the survey. Participants could complete the paper-based survey at the time they were invited or leave their e-mail address to complete an electronic copy. Reminders to complete the electronic copy were sent 1 month later (6 dentists left their e-mail addresses and 2 returned completed digital surveys). Participation in this study was voluntary and ethics approval was obtained from The University of Texas Health Science Center at Houston Committee for the Protection of Human Subjects (HSC-DB-14-0382). Paper-based surveys were anonymous, and investigators kept confidential the e-mail addresses that were collected for completion of the digital survey.

### 2.2. Survey Design

The authors (dentist geriatrician, neuroscientists) created a structured questionnaire consisting of 42 questions, which contained both closed and open-ended questions. The questionnaire was designed to understand dentists’ barriers and motivations, referral strategies, and treatment practices for patients with movement disorders. Three dental professionals, a neurologist, and a neuroscientist were consulted to establish the face and content validity of the survey. In the design of the survey, we included two sets of questions that asked about the same concept in two different ways. We checked survey reliability by quantifying the agreement of respondents’ answers to each question pair. The first question pair had a point biserial correlation of r = 0.252, *p* = 0.007 and the second question pair (with dichotomous answer choices) had a phi coefficient of phi = 0.24, *p* < 0.05. Thus, both question pairs had significant correlation, indicating reliability. Questions in the survey were divided into four domains: professional background (5 questions, Q1 to Q5), referral strategies, including barriers and motivators to providing care (20 questions, Q6 to Q25; the first 5 questions determined if the participant had experience treating patients with movement disorders and those who did not were told to skip to the demographic questions), treatment strategies (13 questions, Q26 to Q38), and demographics (4 questions, Q39 to Q42). The survey consisted of multiple-choice questions, Likert-type charts, open-ended questions, and short clinical scenarios to evaluate referral strategies. Here, we report the results regarding barriers and motivations for dentists to treat patients with movement disorders. Participants were asked to select all barriers or motivators that apply.

### 2.3. Data Analysis

A sample size calculation was conducted, estimating that 15,872 dentists work in the state of Texas [21]. Although 1.5% of people age 50+ in the U.S. have movement disorders, no studies have estimated the number of dental professionals that treat this patient population. To estimate a dentist population proportion with an alpha = 0.05 and power = 0.80, the projected sample size needed is approximately *n* = 163 with this effect size.

Descriptive statistics for participants’ demographics, professional backgrounds, and barriers and motivations for treating patients with movement disorders were calculated as frequencies and percentages of included survey responses.

A Chi-squared (χ^2^) test was used to assess the differences in all categorical data between groups, namely dentists who do or do not treat patients with movement disorders. A *p*-value less than 0.05 was considered statistically significant. To determine which barriers or motivators were statistically different from the others, a post hoc z-test was performed on the adjusted residuals with Bonferroni correction.

## 3. Results

### 3.1. Participants’ Demographics and Professional Backgrounds

A total of 188 of the invited 253 dentists completed the survey (six dentists left their e-mail addresses and two returned completed digital surveys), for a 74% response rate. Of these, 12 surveys were excluded for not meeting inclusion criteria (Figure 1).

Table 1 displays that, of the surveys included, 65.9% (*n* = 116) of dentists reported treating adult patients with movement disorders; of these, most saw fewer than 10 patients with movement disorders per month (87.9%). Seventy percent of respondents were male, reflecting the gender gap seen in dentists in the U.S. (21). More than half of the participants were over 50 years of age (58.5%), and the majority of respondents were white (60.2%), followed in occurrence by Hispanic/Latino professionals (15.9%). When the two groups (dentists who have or have not treated patients with movement disorders) were compared, there were no statistically significant differences in the frequencies observed for gender, age, or race/ethnicity (X^2^ (1, *N* = 172) = 1, *p* = 0.3; X^2^ (1, *N* = 172) = 1.5, *p* = 0.2; X^2^ (6, *N* = 176) = 11.6, *p* = 0.07, respectively). However, considering all participants, there is a higher prevalence of dentists who work at private or small practices and our sampling included very few participants from hospital, university, or big practice settings.

All included respondents had a DDS degree and 81.3% of them had more than 10 years of experience practicing dentistry (Table 1). Only 38% of respondents had completed continuing education regarding dental care of older adults; 8.5% took continuing education courses about dental treatment of patients with a neurological disorder and 6.8% had continuing education specific to dental care of patients with movement disorders. The majority of respondents worked in an individual practice (68.2%) or small group practice (19.3%). Of the 14 participants who reported treating more than 10 patients with movement disorders per month, four belonged to a large group practice, hospital, or dental school, whereas 10 had an individual practice or belonged to a small group practice. When the two groups (dentists who treat and dentists who do not treat patients with movement disorders) were compared, there were no statistically significant differences in the frequencies observed for continuing education, years of experience, or type of practice (X^2^ (2, *N* = 94) = 0.3, *p* = 0.9; X^2^ (2, *N* = 174) = 3.9, *p* = 0.13; X^2^ (5, *N* = 175) = 1.4, *p* = 0.9, respectively).

### 3.2. Barriers to Treating Patients with Movement Disorders

**Figure 2:**
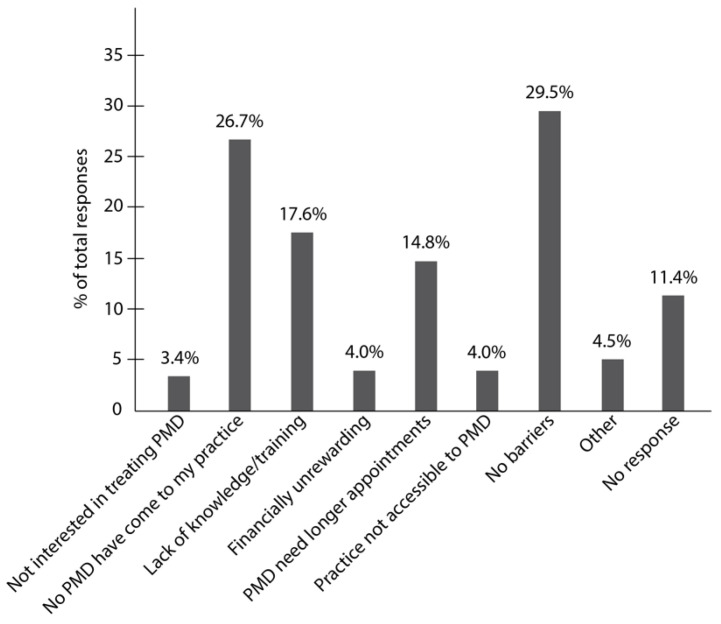
Barriers to treating patients with movement disorders reported by dentists in Texas. Participants reported having no barriers to treating patients with movement disorders (29.5%) and that no patients with these disorders have visited their practice (26.7%). The barriers most often reported by dentists were lack of education specific for this patient population (17.6%) and patients with movement disorders needing longer appointments than other patients (14.8%). Few respondents reported not being interested in treating this patient population (3.4%), that treating them is unrewarding financially (4.0%), or that their practice is not accessible (4.0%). PMD: patients with movement disorders.

Respondents most commonly reported no barriers to treating adult patients with movement disorders (29.5%) or that no patients with these disorders have visited their practice (26.7%) (Figure 2). The next most prevalent barriers reported were lack of knowledge and training specific for this patient population (17.6%) and the need of patients with movement disorders to have longer appointments than other patients (14.8%) (Figure 2). A minority of respondents reported not being interested in treating this patient population (3.4%), that treating them is unrewarding financially (4.0%), or that their practice is not accessible to patients with movement disorders (4.0%) (Figure 2). A few respondents (4.5%) wrote a comment on the “Other” section of the survey stating that they will not treat a patient if they consider the procedure to be unsafe given the conditions (i.e., if the patient has uncontrollable movements or if the patient needs sedation) (data not shown).

When we compared the responses from dentists who treat patients with movement disorder swith those who do not treat patients with movement disorders, there was a significant difference between the barriers cited by the two groups, (X^2^ (8, *N* = 205) = 54.9, *p* < 0.0001). A post-hoc z-test on the adjusted residuals with Bonferroni correction revealed that dentists who do not treat patients with movement disorders more often reported that no patients with movement disorders have come to their practice (*p* < 0.0001). Dentists who treat patients with movement disorders reported significantly more often to have no barriers (*p* = 0.00016) and that appointments with patients with movement disorders last longer than average (*p* = 0.007) (Table 2).

### 3.3. Motivations to Treat Adult Patients with Movement Disorders

**Figure 3:**
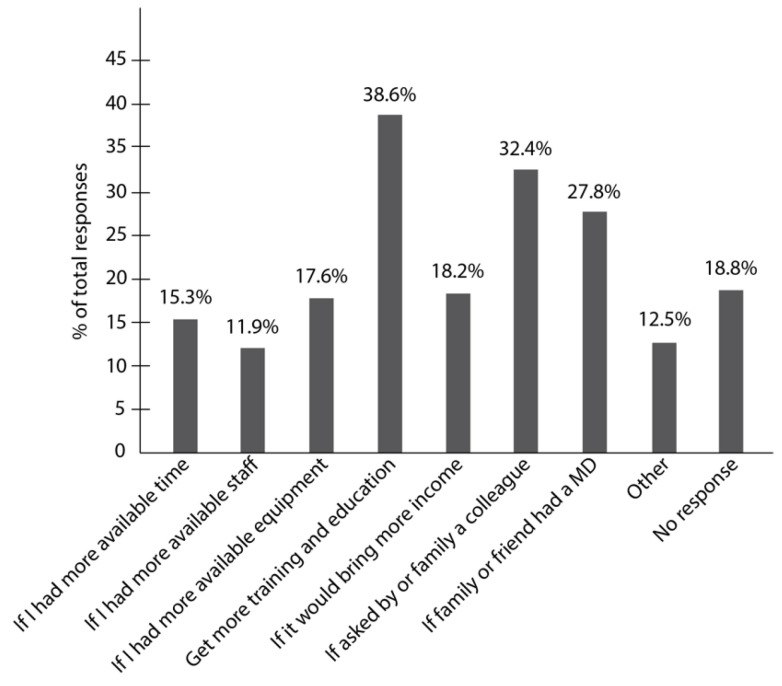
Motivations to treat patients with movement disorders reported by dentists in Texas. The most frequently reported motivations to treat patients with movement disorders were if respondents could have more training and education on the subject (38.6%), if they were asked by a colleague to treat a patient with a movement disorder (32.4%), and if a family member or friend had a movement disorder (27.8%). Other motivations reported less frequently included more available time (15.3%), more available staff (11.9%), more available equipment (17.6%), bringing more income (18.2%), and other (12.5%). MD: movement disorder.

The main motivations reported to treating patients with movement disorders were if dentists could have more training and education on the subject (38.6%), if they were asked by family or a colleague to treat a patient with a movement disorder (32.4%), and if a family member or friend had a movement disorder (27.8%) (Figure 3). Other motivations included more available time (15.3%), more available staff (11.9%), more available equipment (17.6%), and bringing more income to the dentist (18.2%) (Figure 3). Some respondents (12.5%) added in the Comments section of “Other” that they do not need extra motivations and would treat any patient who comes to their practice if it is safe to do so (data not shown).

Dentists who treat patients with movement disorders did not report different motivators by type or frequency than dentists who do not treat patients with movement disorders (Table 3).

## 4. Discussion

Our objective was to investigate the barriers dental professionals perceive when treating patients with movement disorders and explore possible motivations of dentists to treat this population. Our main finding is that most dentists do not perceive barriers to treating patients with movement disorders, rather, dentists report such patients have not visited their practices. Thus, barriers to good oral health for patients with movement disorders may not include unwillingness from the provider, but barriers encountered by the patients. These may include a lack of dental insurance, transportation to the dentist, or a caregiver to assist with oral hygiene [7,14]. Regarding motivations, nearly 40% of oral health professionals said education about the treatment of patients with movement disorders would motivate them to treat more such patients.

One-hundred and seventy-six dentists completed a structured survey and about two-thirds reported treating adult patients with movement disorders; almost 90% of respondents reported treating fewer than 10 patients with movement disorders per month. The low number of patients with movement disorders who go to the dentist leads to fewer opportunities for dentists to gain experience with this population. The number of these patients that dentists reported to treat is distributed in low numbers across dentists rather than grouped with a few providers. Thus, patients may stay with their dentist after diagnosis with a movement disorder as opposed to switching to a dentist with experience in neurological care, as would be the case if cohorts of patients were treated by a few dentists. This information is valuable because it highlights the importance of introducing treatment planning and care for patients with movement disorders—or any special need—in dental schools’ curricula and the need for continuing education on this topic [22].

When we asked dentists what barriers they face when treating adult patients with movement disorders, 29.5% reported no barriers and 26.7% that no patients with movement disorders have visited their practice. We conclude the prevalence of movement disorders is too low for every dentist to have a patient with a movement disorder in their patient family. Likely, patients themselves encounter barriers to going to the dentist, although no research has yet been done to answer this question. Other patient populations have reported physical barriers (i.e., inability to reach the dentist’s office), mental barriers (i.e., anxiety about the dental appointment) or insurance barriers (i.e., Medicare not covering dental care) [15,23,24]. One barrier that was more often reported by dentists who treat patients with movement disorders is that these patients may require longer appointments. This highlights the importance of patient and provider education, for example suggesting that the patient come shortly after their morning dose of medication to avoid tremors and dyskinesia during the appointment [25].

In agreement with the reported barriers, the main motivator to treat a patient with a movement disorder was additional training and education for this unique population (38.6%). Dentists also reported that they would be motivated if a colleague asked them to treat a patient with a movement disorder or family or a friend had a movement disorder (32.4% and 27.8%, respectively). This finding does not necessitate that dentists who do not receive a referral or have personal experience with a movement disorder are biased toward refusing to treat such patients. Rather, respondents who had not treated patients with movement disorders stated these patients had not attended their clinic. Other important motivations reported included if they had more available time (15.3%), if they had more available staff (11.9%), if they had more available equipment (17.6%), and if it would bring more income to the practice (18.2%). These motivations suggest that dentists may see the treatment of patients with movement disorders as a challenge and that education about strategies to treat these patients may change this perception. Indeed, evidence-based recommendations for patients with PD exist to combat oral conditions like poor oral hygiene, hyposalivation with paradoxical drooling, tooth decay and loss, periodontal disease, temporomandibular disorder, and burning mouth syndrome [26,27,28].

Our study has some limitations. The study design was a structured survey, which gathers subjective data and, as such, conclusions must be taken with this in mind. For example, a number of dentists reported that they have a lack of training and knowledge for this patient population and that more continuing education on the topic would be a motivation. If such education is offered, however, it is unknown if dentists will choose to enroll. The survey was completed by dentists in the state of Texas, making generalization of the results to the rest of the country or the world uncertain. Therefore, more studies are needed to investigate if similar barriers and motivations exist for dentists everywhere. Our sampling strategy aimed to reduce bias by inviting dentists consecutive at local and regional dental meetings, however most participants worked at private or small practices and very few at larger hospital or university-based settings. This is important to note because these results mostly reflect the opinions of small practice dentists. Hospitals and university clinics have facilities to support procedures of greater risk that are often required by patients with complex medical conditions. Thus, dentists who work in larger settings may see a greater number of patients with PD or HD and/or have different opinions. In addition, sampling from continuing education and conference settings may introduce a selection bias for dentists who show a higher interest in continuing their studies or even who could afford to attend these events. Finally, our study only investigated select self-perceived barriers and motivations and more may exist for inclusion in future studies. For example, although many respondents reported their willingness to treat any patient in the “Other” section, we did not directly ask if they had refused to treat a patient with movement disorders or if the dental office is accessible to these patients. These questions would be important to determine if a dentist’s intent could translate to reality.

## 5. Conclusions

In conclusion, our findings suggest that dentists in this sample are willing to receive patients with movement disorders in their practices and therefore, future studies should research barriers and motivators encountered by patients with movement disorders. Dentists also expressed the need for specialized continuing education. Dental schools and professional dental associations should consider offering continuing education courses focused on evidence-based recommendations for treating patients with movement disorders. These courses should not be limited to the movement aspects of these disorders but also include other non-motor symptoms that may affect the oral health of these patients [26,27,28].

## Figures and Tables

**Table 1:** Demographics and professional backgrounds of responding dentists.

		All (*n* = 176)	Have Treated PMD (*n* = 116)	Have Not Treated PMD (*n* = 60)
Gender *N* (%)				
	Female	48 (27.3)	29 (25.0)	19 (31.7)
	Male	124 (70.4)	85 (73.3)	39 (65.0)
	NR	2.3 (4.0)	1.7 (2.0)	3.3 (2.0)
Age *N* (%)				
	21–50 years	69 (39.2)	42 (36.2)	27 (45.0)
	>50 years	103 (58.5)	72 (62.1)	31 (51.7)
	NR	4 (2.3)	2 (1.7)	2 (3.3)
Race/Ethnicity *N* (%)				
	White	106 (60.2)	77 (66.4)	29 (48.3)
	White Hispanic/Latino	28 (15.9)	11 (9.5)	17 (28.3)
	Black	7 (4.0)	5 (4.3)	2 (3.3)
	Black Hispanic/Latino	3 (1.7)	2 (1.7)	1 (1.7)
	Asian	25 (14.2)	17 (14.7)	8 (13.4)
	Other	3 (1.7)	2 (1.7)	1 (1.7)
	NR	4 (2.3)	2 (1.7)	2 (3.3)
Continuing Education *N* (%)				
	Geriatric Dentistry	67 (38.0)	49 (42.2)	18 (30)
	Neurology Patient Dentistry	15 (8.5)	10 (8.6)	5 (8.3)
	PMD Dentistry	12 (6.8)	9 (7.8)	3 (5.0)
Years of experience *N* (%)				
	<5 Years	24 (13.6)	12 (10.3)	12 (20.0)
	6–10 Years	7 (4.0)	4 (3.5)	3 (5.0)
	>10 Years	143 (81.3)	100 (86.2)	43 (71.7)
	NR	2 (1.1)	0	2 (3.3)
Type of practice *N* (%)				
	Hospital based	4 (2.3)	3 (2.6)	1 (1.7)
	University based	4 (2.3)	2 (1.7)	2 (3.3)
	Individual	120 (68.2)	80 (69.0)	40 (66.7)
	Small group practice	34 (19.3)	22 (19.0)	12 (20.0)
	Large group practice	7 (3.9)	5 (4.3)	2 (3.3)
	Other	6 (3.4)	3 (2.6)	3 (5.0)
PMD per month *N* (%)				
	<10		102 (87.9)	
	11–25		12 (10.3)	
	>25		2 (1.7)	

PMD: Patients with movement disorders; NR: No response.

**Table 2:** Differences reported in barriers to treat patients with movement disorders between dentists who have or have not treated this patient population, *N* (%).

	Have Treated PMD (*n* = 116)	Have Not Treated PMD (*n* = 60)
I am not interested in treating this patient population	4 (3.4)	2 (3.3)
No PMD have come to my practice	14 (12.1)	33 (55.0) *
My training is for a different patient population	19 (16.4)	12 (20.0)
It is financially unrewarding	7 (6.0)	0
Patient appointments are longer than average	25 (21.6)	1 (1.7) *
My practice is not accessible to PMD	5 (4.3)	2 (3.32)
No barriers	46 (39.7)	6 (10.0) *
Other	6 (5.2)	3 (5.0)
None marked	13 (11.2)	7 (11.7)

PMD: Patients with movement disorders; Chi-squared post hoc z-test on adjusted residuals with Bonferroni correction, * *p* < 0.05.

**Table 3:** Differences reported in the motivations to treat patients with movement disorders between dentists who have or have not treated this patient population, *N* (%).

	Have Treated PMD (*n* = 116)	Have Not Treated PMD (*n* = 60)
If I had more available time	19 (16.4)	8 (13.3)
If I had more available staff	13 (11.2)	8 (13.3)
If I had more available equipment	21 (18.1)	10 (16.7)
If I had the time to get training and education on the subject	45 (38.8)	23 (38.3)
If it would bring more income to my practice	26 (22.4)	6 (10)
If I were asked by a colleague to treat a PMD	44 (37.9)	13 (21.7)
If a family member or friend of mine had a MD	37 (31.9)	12 (20)
Other	17 (14.7)	5 (8.3)
None marked	17 (14.7)	16 (26.7)

PMD: Patient with a movement disorder; MD: Movement disorder.

## Data Availability

The data that support the findings of this study are available from the corresponding author upon reasonable request.
